# Integrated analysis of multifactorial stress combination impact on citrus plants

**DOI:** 10.1007/s00425-025-04866-z

**Published:** 2025-11-05

**Authors:** Lledó Rodríguez-Azorín, Aurelio Gómez-Cadenas, María F. López-Climent, Vicente Vives-Peris

**Affiliations:** Department of Biology, Biochemistry and Natural Sciences, Universitat Jaume I, Avda. Sos Baynat s/n, Valencian Community, Castelló de La Plana, 12071 Castelló, Spain

**Keywords:** Abiotic stress, Drought, Heat stress, Nutritional deficiency, Water stress

## Abstract

**Main conclusion:**

Multifactorial stress combination negatively affects citrus performance, especially when these plants are under three or more stresses, affecting citrus growth at different levels including phenotypic, physiological, biochemical and molecular levels.

**Abstract:**

In nature, biotic and abiotic factors affect plant growth and development. “Multifactorial Stress Combination” (MFSC) refers to situations in which three or more stressors occur simultaneously or sequentially on plants. Its importance lies in the drastic reduction in plant survival under such complex stress scenarios. In this work, we studied the effect of five stresses and their combinations (deficiencies in nitrogen, phosphorus and potassium with water and heat stress) on Carrizo citrange, a citrus genotype widely used in physiological studies. Nutrient deficiencies were applied for three months using specific irrigation solutions. To impose heat stress, plants were maintained for three days in environmental chambers set at 24 °C (control) and 40 °C (heat), while drought was simulated by transferring them to dry perlite. MFSC clearly impacted plant phenotype, increasing leaf damage and decreasing shoot weight, particularly under three or more stressors. Gas exchange parameters and total pigment content were only affected under the combination of four or five stressors, respectively. Oxidative damage increased in plants subjected to five stresses, as indicated by increased malonaldehyde content. A progressive rise was observed in abscisic acid, jasmonic acid, salicylic acid, phaseic acid and indole-3-acetic acid as stress complexity increased, highlighting their involvement as key regulators of the plant stress response. The observed upregulation of galactose metabolism suggested an alternative pathway for energy production and sugar accumulation as essential responses to a complex stress scenario. Overall, results demonstrate the severe impact of MFSC on citrus development, with plant damage increasing exponentially under three or more stressors.

**Supplementary Information:**

The online version contains supplementary material available at 10.1007/s00425-025-04866-z.

## Introduction

Plants are constantly exposed to a wide variety of biotic and abiotic stresses. In recent decades, climate change has enhanced both the severity and frequency of these harmful conditions, particularly abiotic stresses such as extreme temperatures, high salinity and drought (Zandalinas et al. 2018b). The hazardous effects of these conditions lead to substantial economic losses in agriculture (Vives-Peris et al. 2023). Increasing attention has been directed in recent years toward understanding the effect of stress combination, ranging from two to six simultaneous stresses. It has been demonstrated that the accumulation of stressors decreases plant growth and yield (Zandalinas et al. 2021).

Multifactorial Stress Combination (MFSC) is defined as the simultaneous or sequential co-occurrence of three or more stressors (Pascual et al. 2023). Studying MFSC is crucial, as plant growth and survival can decrease drastically with the increasing number of combined stresses, even when each individual stressor has little or no effect acting individually (Zandalinas and Mittler 2022; Balfagón et al. 2023). Moreover, plant responses to combined stresses are unique and cannot be extrapolated from the responses to individual stressors (Zandalinas et al. 2021). MFSC has been studied in species such as Arabidopsis (Zandalinas et al. 2021), rice (Sinha et al. 2024), maize (Sinha et al. 2024), and soybean (Peláez-Vico et al. 2024). However, studies on MFSC in woody species remain scarce, and research on citrus has been limited to combinations involving no more than three stress conditions (Balfagón et al. 2023).

Citrus have been present in the Mediterranean basin for centuries. Species such as orange, mandarin or lemon trees have found in these regions edaphic and climatic conditions which allow them to produce high-quality fruit, which are consumed worldwide, having an important impact on the global economy (Duarte et al. 2016). Citrus responses to water and heat stress, combined or individually, have been extensively studied, since these conditions are very exacerbated by climate change (Zandalinas et al. 2017, 2018b; Balfagón et al. 2022a, b). When plants face water stress alone, stomatal closure is triggered, leading to reduced photosynthetic activity and a consequent decline in photosystem II quantum yield ($\Phi$PSII), with the extent of the effect varying by species and stress intensity (Zandalinas et al. 2016b). However, under heat stress, the stomatal response is typically the opposite, leading to stomatal opening. As a result, photosynthetic activity is frequently higher under heat stress and may even have a positive effect on $\Phi$PSII, again depending on the species and stress intensity. When both stresses occur simultaneously, the physiological effects associated with water limitation generally outweigh those triggered by heat, leading to a marked reduction in photosynthetic activity in citrus plants (Zandalinas et al. 2016b).

Phytohormones have been demonstrated to play a pivotal role in plant responses to abiotic stress conditions. Among them, abscisic acid (ABA), jasmonic acid (JA), salicylic acid (SA) and indole-acetic acid (IAA) have been described as signaling molecules in different stress tolerance processes in citrus plants subjected to abiotic stress conditions (Vives-Peris et al. 2017). In response to MFSC, phytohormone content and biosynthetic pathways are significantly altered, contributing to the regulation of plant adaptation mechanisms under adverse conditions (Pascual et al. 2023).

In agriculture, fertilizers based on nitrogen, phosphorus and potassium are widely used, as these macronutrients are essential for optimal plant growth and development (Kulcheski et al. 2015). Nitrogen, an integral component of nucleic acids and proteins, is mainly absorbed in the form of nitrate (NO_3_^−^) and ammonium (NH_4_^+^) ions via specific membrane transport proteins (de Bang et al. 2021). After uptake, nitrate is reduced to nitrite and subsequently to ammonium via nitrate reductase and nitrite reductase enzymes. The resulting ammonium is then assimilated into organic molecules, mainly glutamine and glutamate, through the glutamine synthetase–glutamate synthase pathway. These amino acids serve as key nitrogen donors for the synthesis of proteins and other nitrogen-containing compounds (Islam et al. 2020). A significant proportion of assimilated nitrogen is allocated to the chloroplasts where it contributes to the formation of essential photosynthetic components such as RuBisCO, photosystem proteins and chlorophyll molecules. Nitrogen can also be metabolized or translocated to developing tissues that act as nitrogen sinks (de Bang et al. 2021). Nitrogen deficiency decreases plant biomass, lowers chlorophyll content and disrupts both photorespiration and photosynthesis. It also causes the accumulation of reactive oxygen species (ROS), triggering oxidative damage in plant cells (Xue et al. 2022).

Phosphorus is a structural component of nucleic acids and membrane phospholipids. This element is also present in molecules related to energy metabolism, such as adenosine triphosphate (ATP) or nicotinamide-adenine dinucleotide phosphate (NADPH). Furthermore, it is crucial in the light-dependent reactions of photosynthesis and in the Calvin-Benson cycle (de Bang et al. 2021). Phosphorus is one of the least available macronutrients in soil. Plants can only absorb it in the form of orthophosphate (Pi), which readily precipitates with cations such as calcium, iron, or aluminium, limiting its bioavailability. To overcome this limitation, plants have developed adaptations for phosphorus uptake, including specific transport proteins such as Phosphate Transporter 1 and Phosphate 1 (Kulcheski et al. 2015). Phosphorus deficiency impairs ATP synthesis, which negatively affects energy-dependent processes such as photosynthesis and carbon assimilation. This results in reduced biomass accumulation and stunted growth. In addition, phosphorus limitation can alter root system architecture as a strategy to improve phosphorus acquisition. It may also delay developmental processes, including flowering, and ultimately reduce crop yield (de Bang et al. 2021).

Potassium is involved in several signaling pathways and it is important for the metabolic adjustment during plant development, reproduction and stress response (Kulcheski et al. 2015). This nutrient activates at least sixty enzymes with important roles in plant growth (Prajapati and Modi 2012). Potassium also plays a key role in regulating stomatal movement. Its uptake by guard cells, accompanied by the movement of counterions, increases cell turgor pressure and promotes stomatal opening (Andrés et al. 2014). Although the underlying mechanisms may vary among species, each plant has developed specific physiological strategies to regulate potassium uptake and maintain ion homeostasis (Kulcheski et al. 2015). Potassium deficiency causes a higher accumulation of ROS, increasing cell oxidative damage. Simultaneously, it induces the synthesis of compatible osmolytes such as proline, soluble sugars, amino acids, and polyols, which contribute to cellular protection under oxidative stress. Additionally, potassium deficiency modifies the phytohormone balance, typically increasing levels of ABA and SA, while reducing the concentration of JA (Xue et al. 2022).

While plant responses to individual and combined deficiencies of essential nutrients such as nitrogen, phosphorus, potassium, sulphur or iron, have been extensively studied (Rietra et al. 2017; Kumar et al. 2021), research examining the interplay between nutrient deficiencies and other abiotic stress factors remains particularly scarce, highlighting the need for more integrative studies in this field. The main objective of this work is to determine the stress response of citrus to MFSC, specifically combining nutritional deficiencies with drought and heat stress. For this purpose, plants of Carrizo citrange, a citrus genotype widely used for stress experiments, were subjected to the combination of the stress conditions mentioned above. Plant responses were evaluated based on a set of phenotypic, physiological, biochemical and molecular parameters.

## Materials and methods

### Plant material and growth conditions

Six-month-old Carrizo citrange (*Citrus sinensis* L. Osbeck x *Poncirus trifoliata* L. Raf.) certified plants were provided in seed trays by an authorized nursery (Beniplant, Peñíscola, Castelló, Spain). Upon arrival, plants were pruned at 35 cm and transferred individually to 10 × 10 × 10 cm black polystyrene pots filled with a 2:1 peat:perlite mixture as substrate. Plants were acclimated in a greenhouse under controlled conditions (50–70% relative humidity with natural photoperiod under a light intensity ranging from 50 to 200 W/m^2^ and day/night temperature averaging 30.0 ± 2.0ºC and 22.0 ± 2.0ºC, respectively) placed at Universitat Jaume I (Castelló de la Plana, Castelló, Spain; 39.991774N, 0.071120W) for two weeks before stress onset.

### Stress treatments and experimental design

**Fig. 1:**
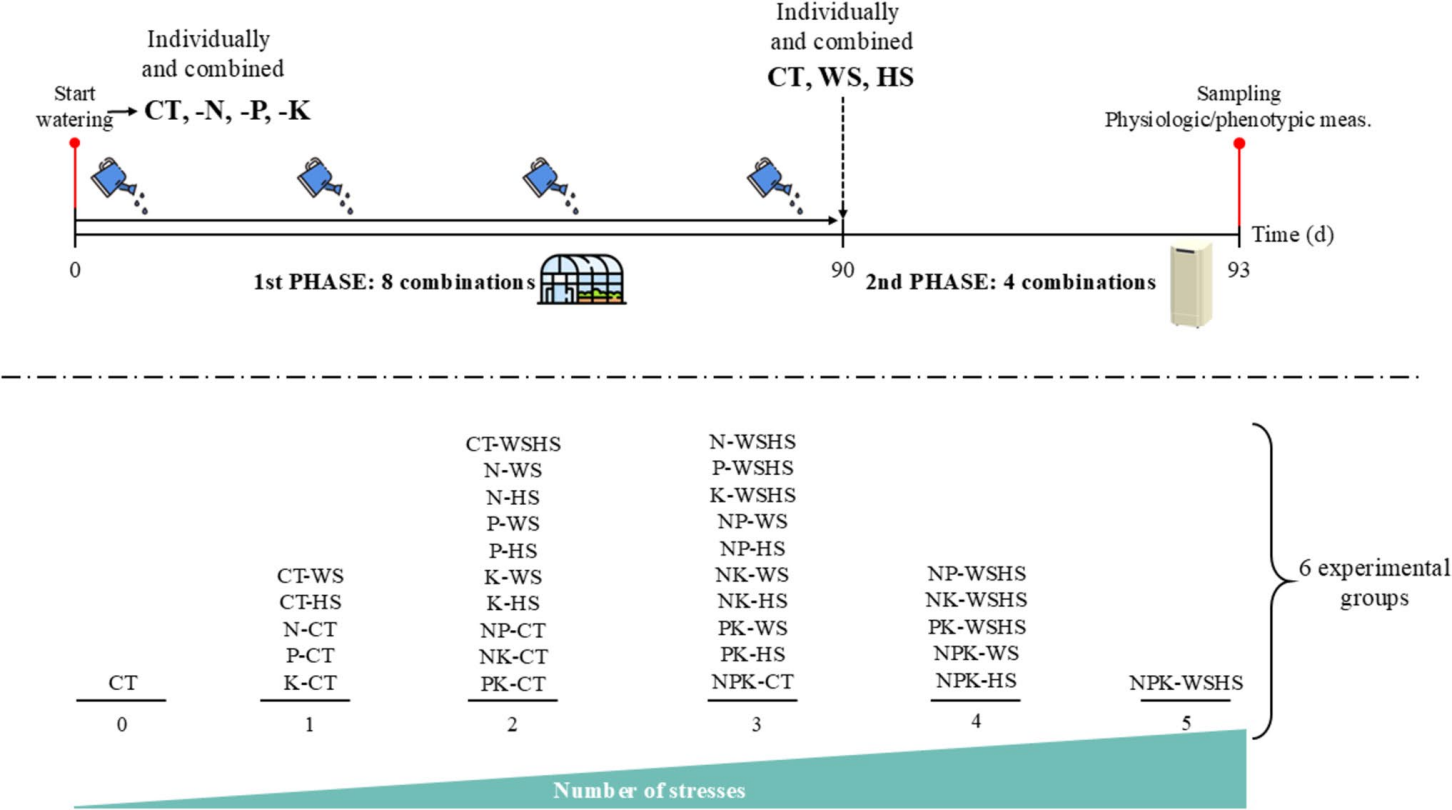
Experimental design to study Carrizo citrange (CC) response to Multifactorial Stress Combination. Nutritional deficiency [nitrogen (N), phosphorus (P) and potassium (K)], water stress (WS) and heat stress (HS) were applied in combination of up to five simultaneous stresses using a two-phase experimental setup. In Phase 1, plants were irrigated for three months under controlled greenhouse conditions with specific irrigation solutions based on the corresponding stress combination: control (CT), nitrogen deficiency (N), phosphorus deficiency (P), potassium deficiency (K), and their combinations (NP, NK, PK, and NPK). Each treatment included 16 plants. In Phase 2, plants from each Phase 1 condition were transferred to environmental chambers and divided into four groups for three days, corresponding to the combinations of the second phase: control (CT), water stress (WS), heat stress (HS), water and heat stress (WSHS). Water stress was induced by transferring the corresponding plants to dry perlite (WS and WSHS) instead of wet perlite (CT and HS); heat stress was induced by setting the chambers at 40ºC (HS and WSHS) instead of 24ºC (CT and WS). Overall, six experimental groups were defined based on the number of combined stresses. The experiment was performed in triplicate

Plants were exposed to MFSC, including nutritional deficiencies (nitrogen, phosphorus, and potassium), water and heat stress conditions. These stresses were applied in two distinct phases: first, nutritional starvation was induced for three months under greenhouse-controlled conditions as described in the previous paragraph. Then, these plants were subjected to water stress, heat stress and their combination in environmental chambers. Control groups without stress conditions’ exposition were included in both phases of the experiment (Fig. 1).

For the first phase, plants were transferred from peat to perlite as an inert substrate and after two days of acclimation plants were divided into eight treatment groups according to the conditions of the first stage including sixteen plants per treatment: control (CT), nitrogen deficiency (N), phosphorus deficiency (P), potassium deficiency (K), nitrogen and phosphorus deficiency (NP), nitrogen and potassium deficiency (NK), phosphorus and potassium deficiency (PK) and nitrogen, phosphorus and potassium deficiency (NPK). Nutritional deficiencies were induced by modifying the macronutrient composition of the irrigation solutions, which were prepared from a 10X concentrated stock using the control irrigation solution described in Arbona et al. (2006), adding the commercial compound Nutrishell (Zenagro, Valencia, Spain) for the micronutrient input (irrigation solution compositions detailed in Online Resource S1). Plants were watered depending on demand, approximately three times a week watering each plant with 100 mL. The final pH was adjusted to 6.0 ± 0.2 at the watering moment adding β-(N-morpholino)-ethanesulphonic acid (MES) at a final 5 mM concentration.

After three months, plants from each nutritional deficiency group were divided into four new treatment groups [control (CT), water stress (WS), heat stress (HS) and combination of water and heat stress (WSHS)] and moved to environmental chambers (Snijders ECD01E), including four plants per treatment (128 plants in total) to apply the stresses of the second phase for three days. To induce water stress, plants included in WS and WSHS conditions were transplanted to dry perlite without irrigation, while plants subjected to CT and HS conditions were transplanted to wet perlite and watered through the rest of the experiment. To induce heat stress, two environmental chambers were set at different temperatures, 24ºC (CT and WS) and 40ºC (HS and WSHS) to maintain the control and heat stress conditions, respectively, both with a 16 h photoperiod. The fourth day, different parameters detailed below were measured, and leaf tissue was collected (excluding the necrotic leaves) and immediately frozen with liquid nitrogen for the corresponding analytical determinations.

The thirty-two stress combinations, which are detailed in Online Resource S2, are classified in six study groups according to the number of applied stress conditions (Fig. 1). This experimental design was based on previous works by Muñoz-Espinoza et al. (2015) and Pascual et al. (2023). The experiment was repeated three times.

### Phenotypic parameters

Phenotypic parameters, including shoot fresh weight and leaf damage, were measured in all groups of plants on the sampling day. Moreover, a picture of the most representative plant of each stress combination was taken. Leaf damage was annotated based on a scale from zero to five, being “zero” non-damaged and “five” the most leaf damage observed (Online Resource S3). The degree of leaf damage of the leaves included in the intermediate third of the stem of each plant was assessed depending on the ratio between undamaged and damaged leaves and on the abscission level; a value from zero to five was awarded to each individual plant.

### Gas exchange parameters

Gas exchange parameters, both transpiration (E) and stomatal conductance (gs), were measured in all plants from each experimental group between 9 AM and 2.30 PM using a portable photosynthesis system (LI-6800, LI-COR Environmental, NE, USA). Light lamp was established at 1000 µmol m^−2^ s^−1^, air flow 150 µmol mol^−1^, and carbon dioxide (CO_2_) of reference fixed at 400 ppm. One undamaged mature leaf from each plant was measured, obtaining four measures per leaf after instrument stabilization at 1–2 min for each replicate (Zandalinas et al. 2016b).

### PSII quantum yield

PSII quantum yield (ФPSII) was measured simultaneously with gas exchange parameters using a portable fluorometer (FluorPen FP-MAX 100, Photon Systems Instruments, Drasov, Czech Republic). Two undamaged mature leaves from each plant were measured on the adaxial side in each group obtaining one measure per leaf and two measurements per plant (Zandalinas et al. 2016b).

### Chlorophyll and carotenoid analysis

Total leaf chlorophyll and carotenoid content of each studied group was extracted by homogenization of 15–30 mg of fresh frozen tissue with 2 mL of dimethyl sulfoxide (DMSO) incubated at 37⁰C overnight in darkness. The absorbance of the liquid phase of the mixture was measured at 665, 649 and 480 nm with a spectrophotometer (Genesys 180, Thermo Fisher, Waltham, MA, USA) and the chlorophyll A, chlorophyll B, and total chlorophyll and carotenoid content were calculated according to the equations described in Wellburn (1994).

### Oxidative status

Leaf malondialdehyde (MDA) analysis was performed for each experimental group according to the protocol described by Hodges et al. (1999) with some modifications. For the extraction, 100–120 mg of fresh frozen tissue were homogenized with 2 mL of 80% (v/v) absolute ethanol by 30 min of sonication (Elma S30, Elmasonic, Singen, Germany). Samples were centrifuged for 20 min at 2500 g. 800 µL of supernatant were mixed with 800 µL of 20% (v/v) trichloroacetic acid, and another 800 µL of supernatant were mixed with 800 µL of (20% (v/v) trichloroacetic acid + 0.5% (v/v) thiobarbituric acid. Both mixtures of each sample were incubated for 1 h at 90⁰C, cooled down and their absorbance was measured at 532, 440 and 600 nm. Total MDA content was calculated according to the equations described in Zandalinas et al. (2017).

Antioxidant capacity was determined following the protocol of 2,2'-azino-bis(3-ethylbenzothiazoline-6-sulfonic acid) (ABTS) described in Chaves et al. (2020) including some modifications. The reaction solution included 7 mM ABTS and 2.45 mM potassium persulfate (K_2_S_2_O_8_), prepared and kept overnight in the dark at room temperature and diluted with distilled water until it reached absorbance values of 0.7 at 734 nm at the measuring moment. For the extraction, 50 mg of fresh frozen leaf tissue were homogenized with 500 µL of methanol using a ball mill (MillMix20, Domel, Železniki, Slovenija) for 10 min at 17 rps. After 10 min of centrifugation at 21,300 g, 10 µL of the supernatant was added to 990 µL of reaction solution and its absorbance at 734 nm was read at 0, 1, 2 and 3 min, calculating the oxidation inhibition percentage of each sample. This percentage was interpolated in a standard curve prepared with commercial Trolox and the obtained results were expressed as the Trolox equivalent concentration per gram of fresh tissue.

### Phytohormone analysis

Phytohormone extraction and analysis were performed using the protocol described in Durgbanshi et al. (2005), including some protocol modifications described by Vives-Peris et al. (2023). Over 100 mg of fresh frozen tissue were homogenized including 6.25 ng of [^2^H_6_]-ABA and [^13^C]-SA and 2.5 ng of dihydrojasmonic acid (DHJA) and [^2^H_5_]-IAA, 5–7 glass beads and ultrapure water until 2 mL during 10 min at 17 rps in ball mill equipment (MillMix20). After centrifugation, the supernatant pH was adjusted to 2.8–3.2 with 80% (v/v) acetic acid. Then, a double liquid:liquid partition was performed with diethyl ether, recovering and evaporating the organic layer. Samples were resuspended in 10% (v/v) methanol by a 10 min sonication (Elma S30) and filtered. This filtered extract was diluted with 10% (v/v) methanol in a 1:3 ratio. For each sample, 15 µL were injected into an Ultra-High Pressure Liquid Chromatography coupled with Mass Spectrometry (UPLC-MS system) (Xevo TQ-S; Waters, Milford, MA, USA) for analyzing phytohormone content. Chromatographic separation was performed using a reversed-phase C18 column (50 × 2.1 mm, 1.6-μm particle size, Luna Omega, Phenomenex, Torrance, CA, USA) as the stationary phase and a gradient of ultrapure water and acetonitrile (both supplemented with 0.1% (v/v) formic acid) as mobile phases with a fixed flow rate of 0.3 mL min^−1^. Phytohormones were detected using a triple quadrupole mass spectrometer, using the transitions described in Vives-Peris et al. (2023). Results were processed using Masslynx v. 4.2 software (Waters), obtaining the phytohormone quantification by the interpolation of the obtained responses in a calibration curve prepared with commercial hormones and the internal standard used during the extraction.

### Gene expression analysis

Leaf samples from plants included in the control group and the five stresses group were used for RNA-Seq analysis, performing three technical replicates of each group. The analysis was performed by Novogene (UK) Company Limited (25 Cambridge Science Park, Multon Road). RNA quantification was performed with Qubit, selecting samples with at least 400 ng of RNA; RNA integrity number (RIN) was measured with Agilent 2100, discarding samples with less than 4.0 RIN; and the purity of RNA was measured by NanoDrop, selecting samples without degradation and/or contamination based on OD260/280 and OD260/230 ≥ 2.0. Sequencing was performed with NovaSeq PE150 on a NovaSeq X Plus Platform. Reads were obtained from the NovaSeq series SE50/PE50/PE250 platform and aligned to the reference genome. Bioinformatic analysis was also executed by Novogene, including gene expression quantification and enrichment analysis of KEGG pathways of differentially expressed genes. Differentially expressed transcripts were defined as those that have an adjusted *P* value < 0.05.

### Statistical analysis

Statistical analysis was performed with the Infostat v.2020 software by two-way analysis of variance (ANOVA) followed by Tukey post hoc test indicating significant differences at *P* value < 0.05 with different letters; and two-tailed Student’s t-test was performed comparing each group with the control group (zero stresses). Statistically significant differences from Student’s *t* test at *P* value < 0.05, 0.01 or 0.001, were marked with one, two, or three asterisks, respectively. To visualize the multivariate data, a Principal Component Analysis (PCA) was performed using SigmaPlot version 14.0 software (Systat Software, Chicago, IL, USA).

## Results

### Multifactorial stress combination increases leaf damage and decreases shoot biomass in citrus plants

**Fig. 2:**
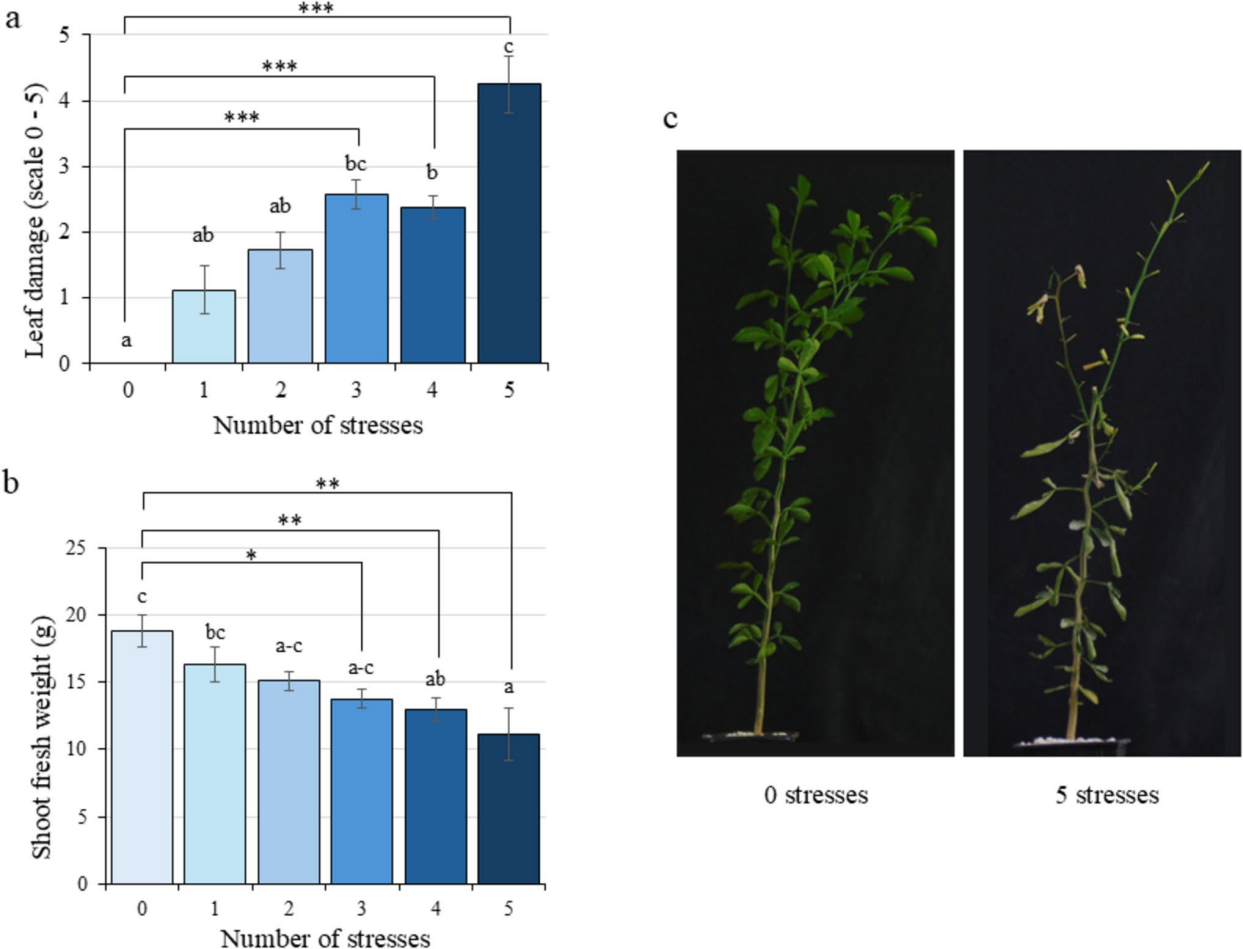
Phenotypic parameters measured on citrus exposed to Multifactorial Stress Combination depending on the number of applied stresses. **a** Leaf damage level on a scale from zero to five. **b** Shoot fresh weight. **c** Pictures of the most representative citrus plant exposed to zero and five stresses combination on the sampling day (*n* = 4). Statistical analysis was performed by ANOVA test using *P* value = 0.05 comparing all the groups simultaneously. Data refer to mean values ± SE. Different letters denote significant differences among groups. An additional statistical analysis was performed by Student’s *t* test. *, significant difference at *P* value < 0.05; **, *P* value < 0.01; ***, *P* value < 0.001

Phenotypic damage was evaluated in each citrus plant at the end of the experiment to compare the incidence of the number of applied stresses, including leaf visual damage level and shoot fresh weight (Fig. 2). Leaf damage level progressively increased with the accumulation of stress factors (Fig. 2a) according to the established damage scale (Online Resource S3). Under control conditions (zero stresses), plants did not exhibit leaf damage. However, when three, four or five stresses were simultaneously applied, significant leaf damage was observed, reaching values of 2.58, 2.38 and 4.25, respectively.

In contrast, shoot fresh weight showed an opposite trend, gradually decreasing as the number of stressful conditions increased. Compared to the control, shoot fresh weight declined by 27.10%, 31.06% and 41.09% under three, four or five combined stress conditions, respectively (Fig. 2b). The increased phenotypic damage associated with a higher number of adverse conditions was clearly evident across treatments (Online Resource S4), with the most representative differences shown between CT-CT and NPK-WSHS conditions (Fig. 2c).

### Multifactorial stress combination reduces ФPSII and chlorophyll content but increases carotenoid accumulation in citrus plants

**Fig. 3:**
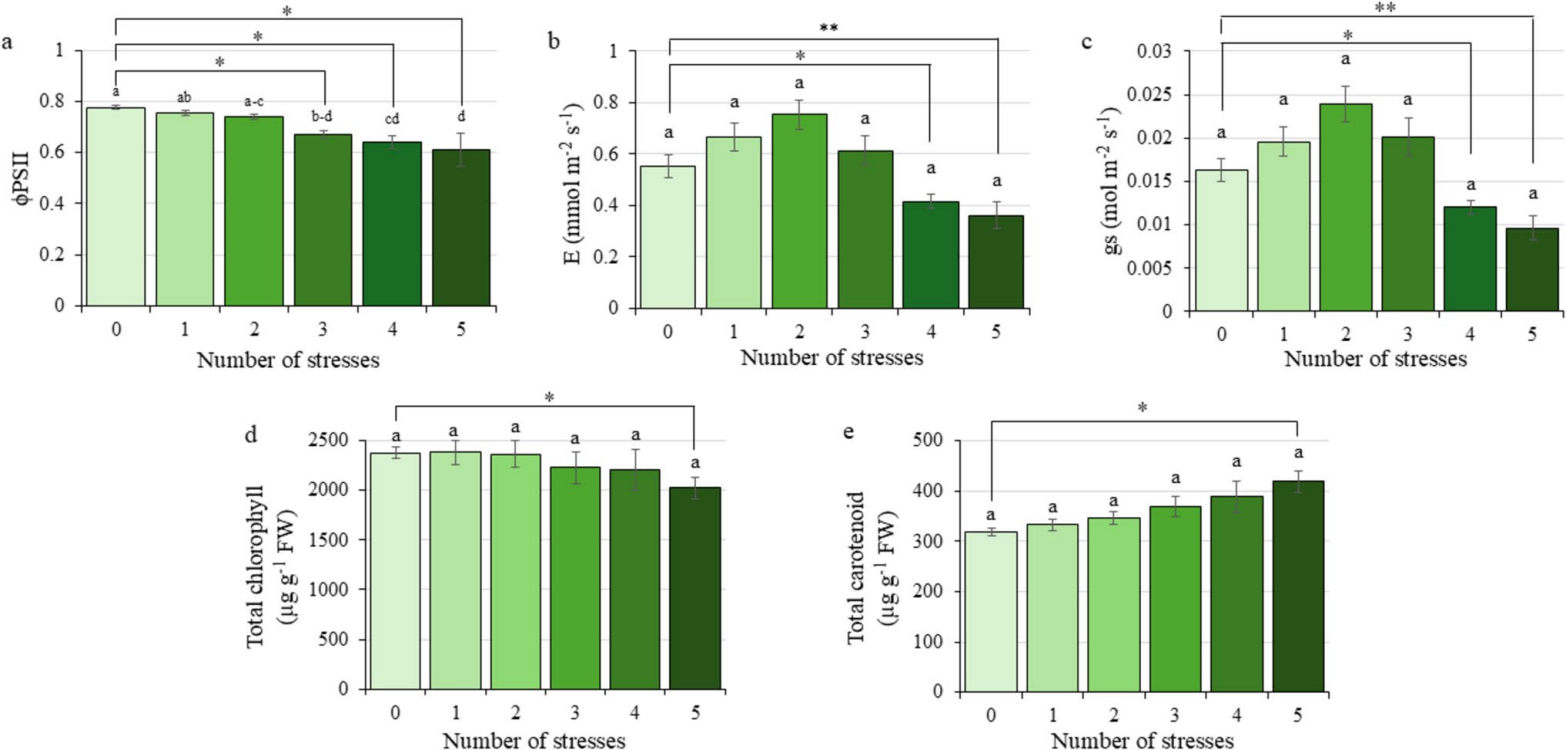
Physiological parameters measured in citrus plants exposed to Multifactorial Stress Combination depending on the number of applied stresses. **a** Photosystem II quantum yield (ФPSII). **b** Transpiration (E). **c** Stomatal conductance (gs). **d** Total chlorophyll content. **e** Total carotenoid content. Data refer to mean values ± SE. Statistical analysis was performed by ANOVA test using *P* value = 0.05 comparing all the groups simultaneously (different letters denote significant differences between groups). An additional statistical analysis was performed by Student’s *t* test. *, significant difference at *P* value < 0.05; **, *P* value < 0.01; ***, *P* value < 0.001

ФPSII, photosynthetic parameters (E and gs), and total chlorophyll and carotenoid content were measured in all the experimental groups (Fig. 3). A progressive decrease in ФPSII was observed with an increasing number of stress factors. Significant reductions of 13.00%, 16.88% and 20.78% in ФPSII values were recorded when plants were subjected to three, four and five simultaneous stresses, respectively, relative to control plants (Fig. 3a).

However, E and gs did not show a linear decreasing trend in response to the number of applied stresses. Instead, their variation appeared to be highly influenced by the type of stress combination due to the wide variation of stress combinations included in these experimental groups (Fig. 1). The different combinations can induce opposite photosynthetic-related responses in plants, leading to an increase in those parameters in some cases against a decrease in them under other stress combinations. Despite that, both parameters showed significantly lower values in plants exposed to four and five stresses compared to the control group, almost independently of the stress combinations, having more importance in the complexity of the stress scenario. Transpiration rate decreased by 25.00% and 34.55% under four and five stresses, respectively, compared to the control situation; gs levels were reduced by 31.25% and 43.75% under the same conditions (Fig. 3b and c).

Chlorophyll A (ChlA) and chlorophyll B (ChlB) contents did not differ significantly among treatments (Online Resource S5). However, total chlorophyll (Fig. 3d) and carotenoid (Fig. 3e) content displayed differences as the number of stresses increased, showing an opposite tendency between them. Chlorophyll content decreased progressively with the increased number of stress factors, while carotenoid levels increased. Specifically, plants subjected to five simultaneous stresses showed a 14.83% reduction in total chlorophyll content and a significant 31.31% increase in carotenoid content, compared to control plants.

### A multifactorial stress combination of five simultaneous stresses triggers oxidative damage and strong antioxidant response in citrus leaves

**Fig. 4:**
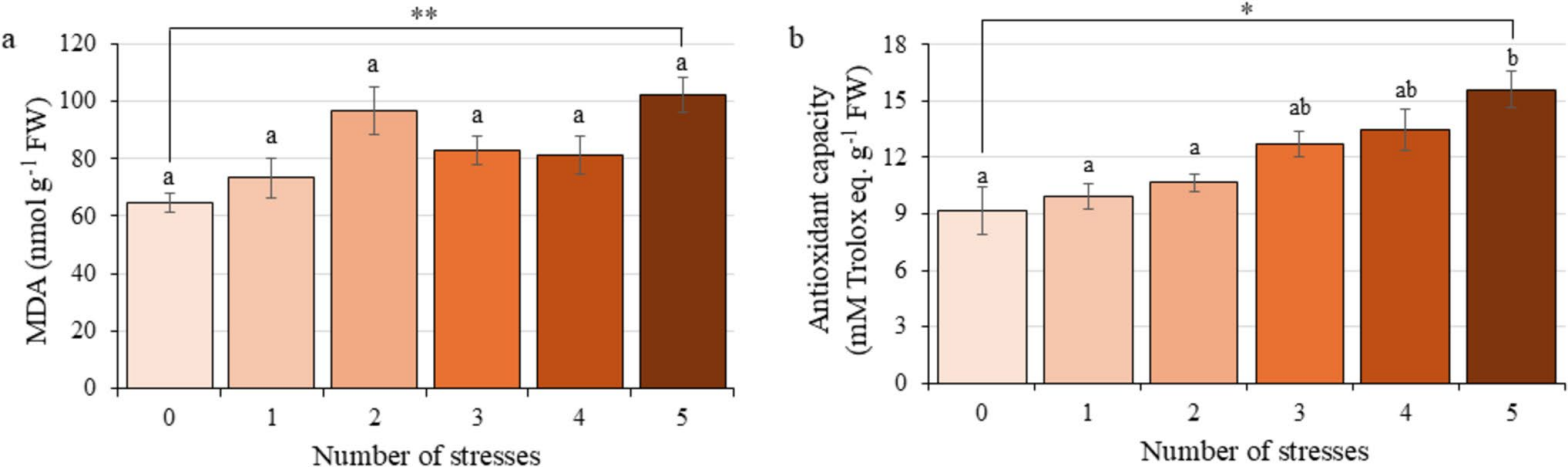
Oxidative status of citrus plants exposed to Multifactorial Stress Combination depending on the number of applied stresses. **a** Malondialdehyde (MDA) leaf content. **b** Antioxidant capacity represented as Trolox equivalents. Statistical analysis was performed by ANOVA test using *P* value = 0.05 comparing all the groups simultaneously. Data refer to mean values ± SE. Different letters denote significant differences between groups. An additional statistical analysis was performed by Student’s *t* test. *, significant difference at *P* value < 0.05; **, *P* value < 0.01; ***, *P* value < 0.001

Leaf MDA content (Fig. 4a) and antioxidant capacity (Fig. 4b) were measured in all groups of plants proposed in the experimental design. The exposure to five stress conditions led to a significant 36.87% increase in MDA content compared to control plants. This oxidative damage increase was accompanied by an enhanced antioxidant capacity, which showed a significant 70.78% rise in comparison to the non-stressed plants.

### Multifactorial stress combination induces accumulation of ABA, PA, SA, JA and IAA in citrus leaves, with highest hormonal responses under five simultaneous stresses

**Fig. 5:**
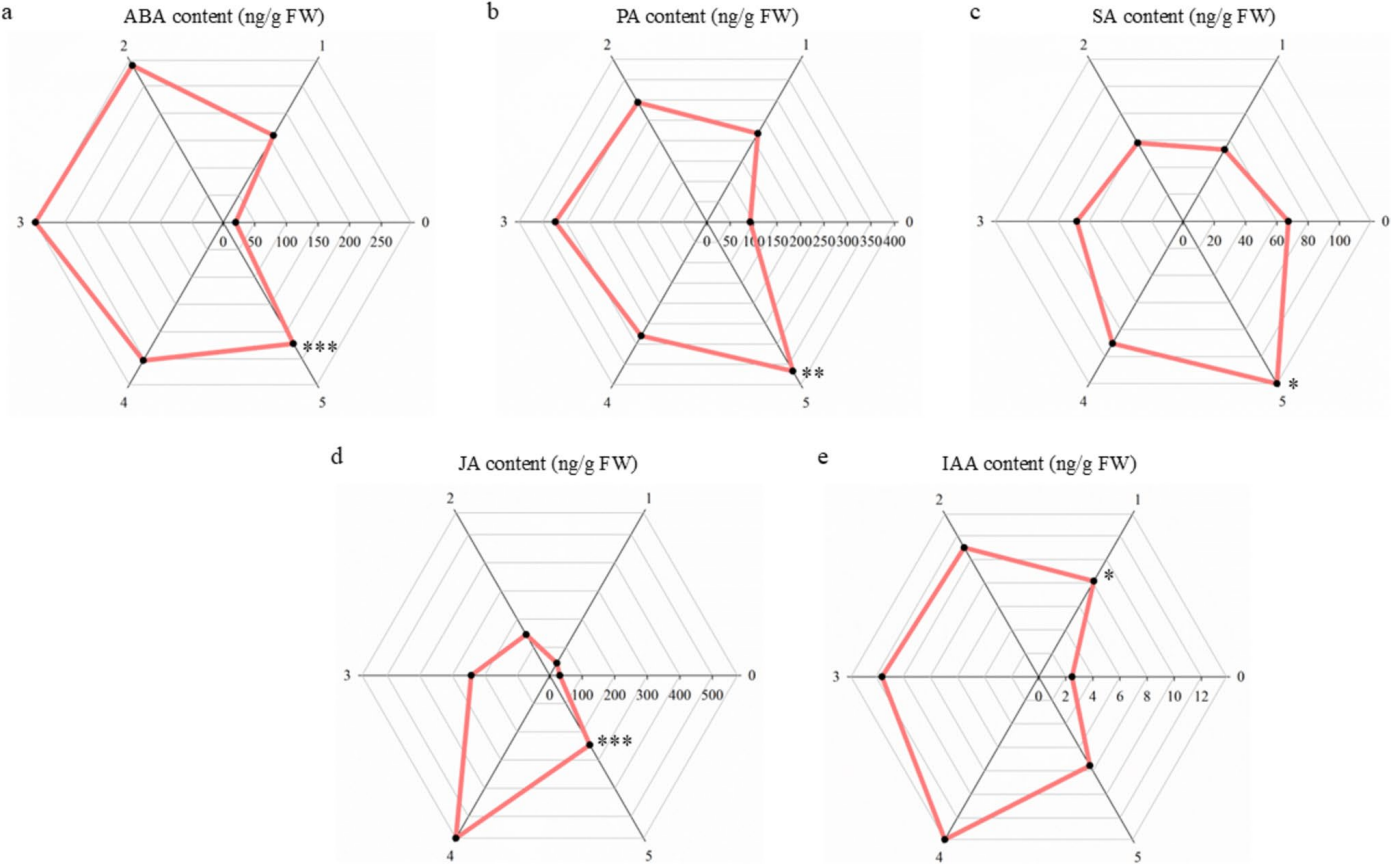
Phytohormonal leaf content of citrus exposed to Multifactorial Stress Combination depending on the number of applied stresses. **a** Abscisic acid (ABA) content. **b** Phaseic acid (PA) content. **c** Salicylic acid (SA) content. **d** Jasmonic acid (JA) content. **e** Indole acetic acid (IAA) content. Data refer to mean values ± SE. Statistical analysis was performed by Student’s *t* test. *, significant difference at *P* value < 0.05; **, *P* value < 0.01; ***, *P* value < 0.001

Leaf phytohormone content, including ABA and its catabolite phaseic acid (PA), SA, JA and IAA, was measured across all experimental groups (Fig. 5). ABA (Fig. 5a), PA (Fig. 5b), SA (Fig. 5c) and JA (Fig. 5d) levels increased progressively with the accumulation of stresses. Under the exposition to five stresses, these hormones increased significantly by 11.25-, 3.97-, 1.78-fold and 7.94-fold, respectively, compared to control plants. Related to JA content (Fig. 5d), the large increase observed in plants subjected to four stress factors was not significant compared to the control. One of the stress combinations included in the four stresses group (PK-WSHS combination) caused a substantial rise in JA concentration not observed in the other combinations included in the same group, contributing substantially to a high variance in JA levels of the group (Online Resource S6).

Regarding IAA content (Fig. 5e), a significant 3.31-fold increase was observed in response to stress. However, no significant differences were detected between control plants and those exposed to two or more stressors, suggesting that IAA accumulation was not proportional to the number of stresses, maybe interfering also with the antagonist relations of IAA with other phytohormones as ABA, JA or SA, or maybe reflecting the priority of accumulating other phytohormones in front of IAA under complex stress scenarios by citrus.

### PCA reveals distinct clustering of control and plants under multifactorial stress combination of five stresses based on phenotypic, photosynthetic, oxidative and hormonal responses

**Fig. 6:**
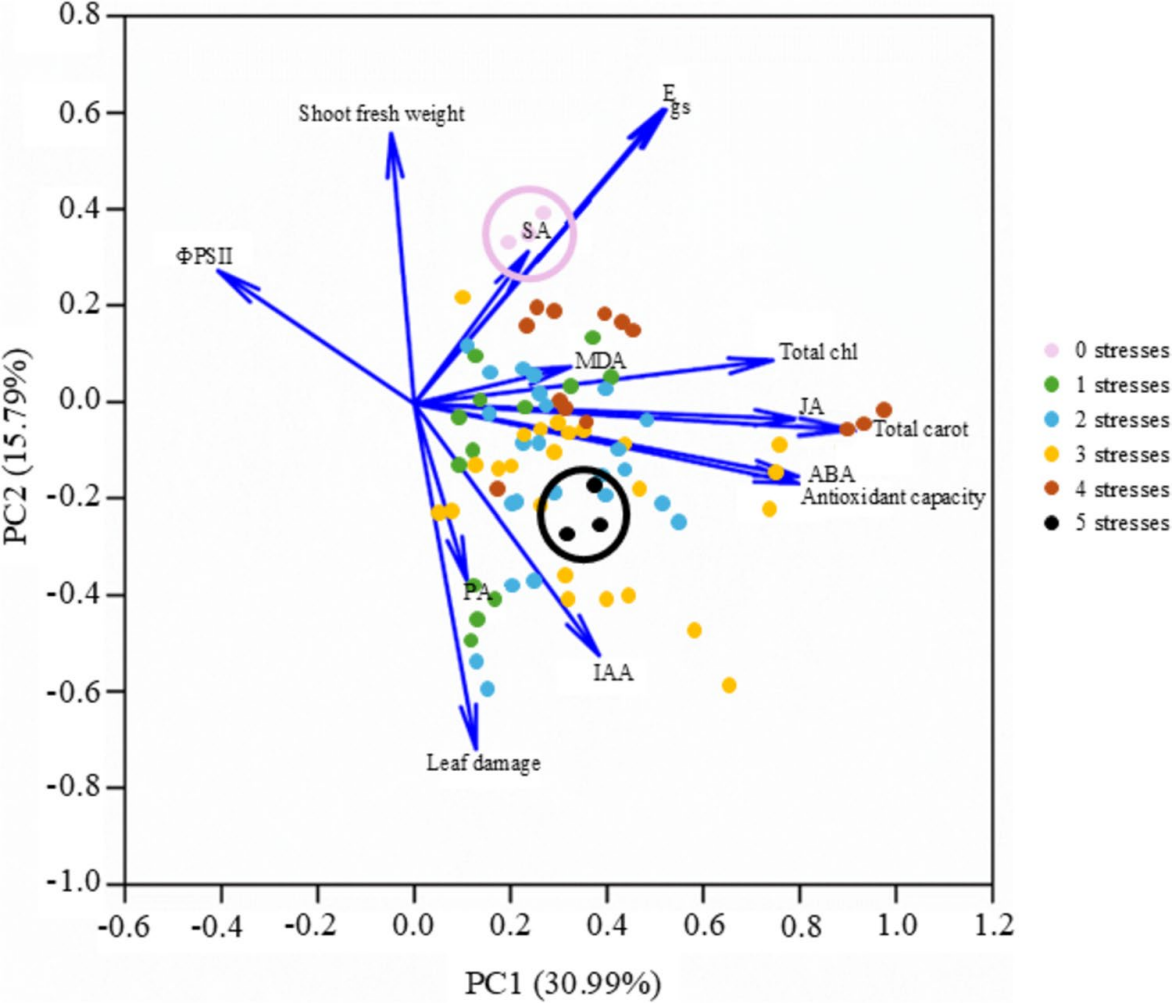
Principal Component Analysis (PCA) plot showing differences in leaf damage, total carotenoids and total chlorophyll content, shoot fresh weight, ФPSII, gs, E, MDA content, antioxidant capacity, ABA content, JA content, SA content, PA content and IAA content of citrus plants exposed to Multifactorial Stress Combination clustering by the number of stresses. Component scores plot is represented by different colour dots

A PCA was performed on the parameters depicted in Figs. 2–5, explaining 46.78% of the total variance (Fig. 6). Principal Component 1 (PC1), accounting for 30.99% of the total variance, was primarily influenced by ABA and JA levels, antioxidant capacity and total chlorophyll and carotenoid content (Online resource S7). In this component, plants under five simultaneous stresses group exhibited higher concentrations of ABA, JA, IAA and PA, whereas control plants showed only baseline levels of these hormones. Similarly, antioxidant capacity was enhanced in the five-stress group, while control plants showed a lower antioxidant activity. Principal Component 2 (PC2), which explained 15.79% of the total variance, was mainly associated with phenotypic traits (leaf damage and shoot fresh weight) as well as the photosynthetic parameters E and gs (Online Resource S7). This component effectively separated control plants from those subjected to five stress factors: the control group showed higher shoot fresh weight, E, and gs, while the five-stress group exhibited increased leaf damage.

No clear separation was observed among the groups subjected to one, two, three and four stresses, in either principal component. This lack of distinct clustering is likely due to the diversity of stress combinations within these groups, each eliciting specific physiological responses, which makes it difficult to cluster them in the PCA.

### RNA-Seq analysis reveals upregulation of key energetic pathways in citrus under complex multifactorial stress combination

**Fig. 7:**
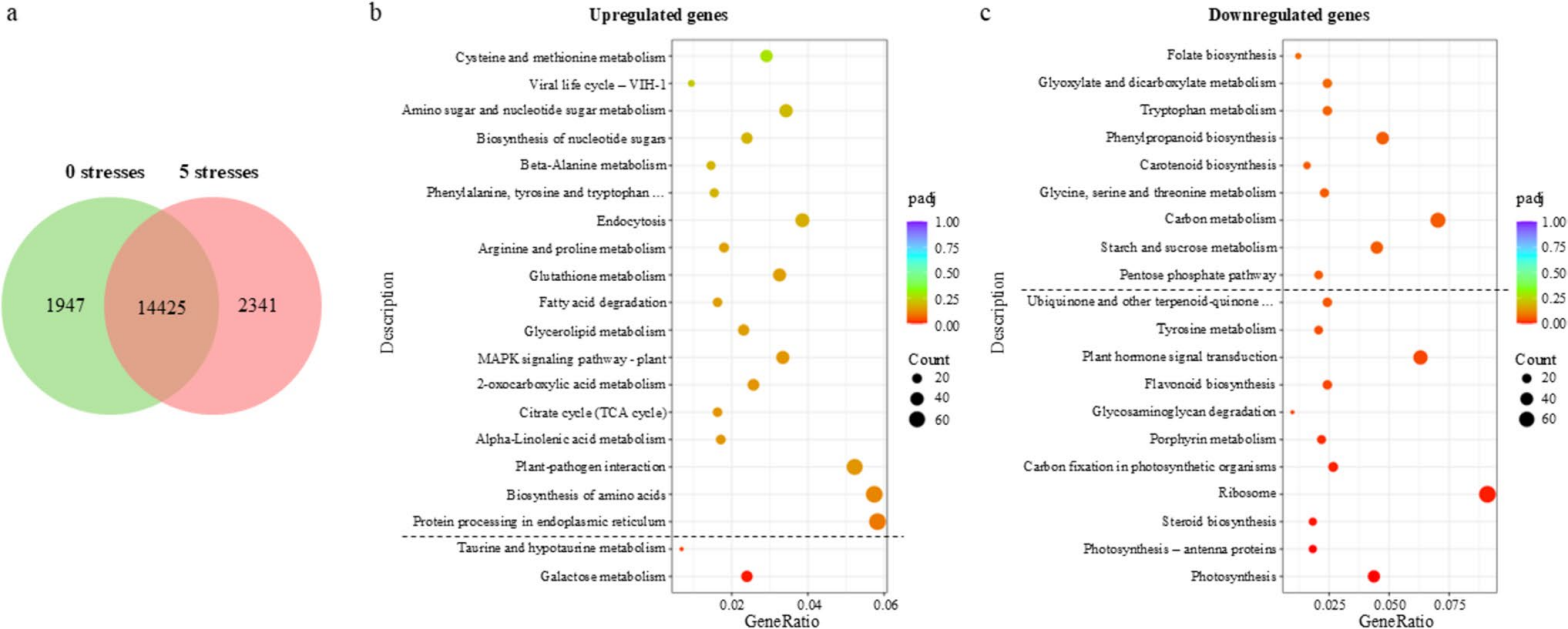
Gene expression analysis of citrus plants exposed to five stresses. **a** Venn diagram showing the overlapping between genes expressed in response to zero (green) and five (red) stresses. **b** Enrichment graph showing upregulated genes included in different metabolic pathways in five stresses group plants compared to control. **c** Enrichment graph showing downregulated genes included in different metabolic pathways in five stresses group plants compared to control. The dashed black line marks the last pathway that contains genes with a statistically significant differential expression based on an adjusted *P* value < 0.05. *Padj* adjusted *P* value

Gene expression analyses of plants from the control group (non-stressed) and those subjected to five simultaneous stress conditions were performed by RNA-Seq (Fig. 7). A total of 14,425 genes were commonly expressed in both groups. However, 1947 genes were uniquely expressed in control plants. Conversely, 2341 genes were exclusively expressed in the five-stress group, resulting in 4288 genes showing a statistically significant differential expression (Fig. 7a). Notably, even among the commonly expressed genes, expression profiles differed markedly between groups. Several metabolic pathways were affected, with specific genes either upregulated (Fig. 7b) or downregulated (Fig. 7c) in response to multifactorial stress.

**Fig. 8:**
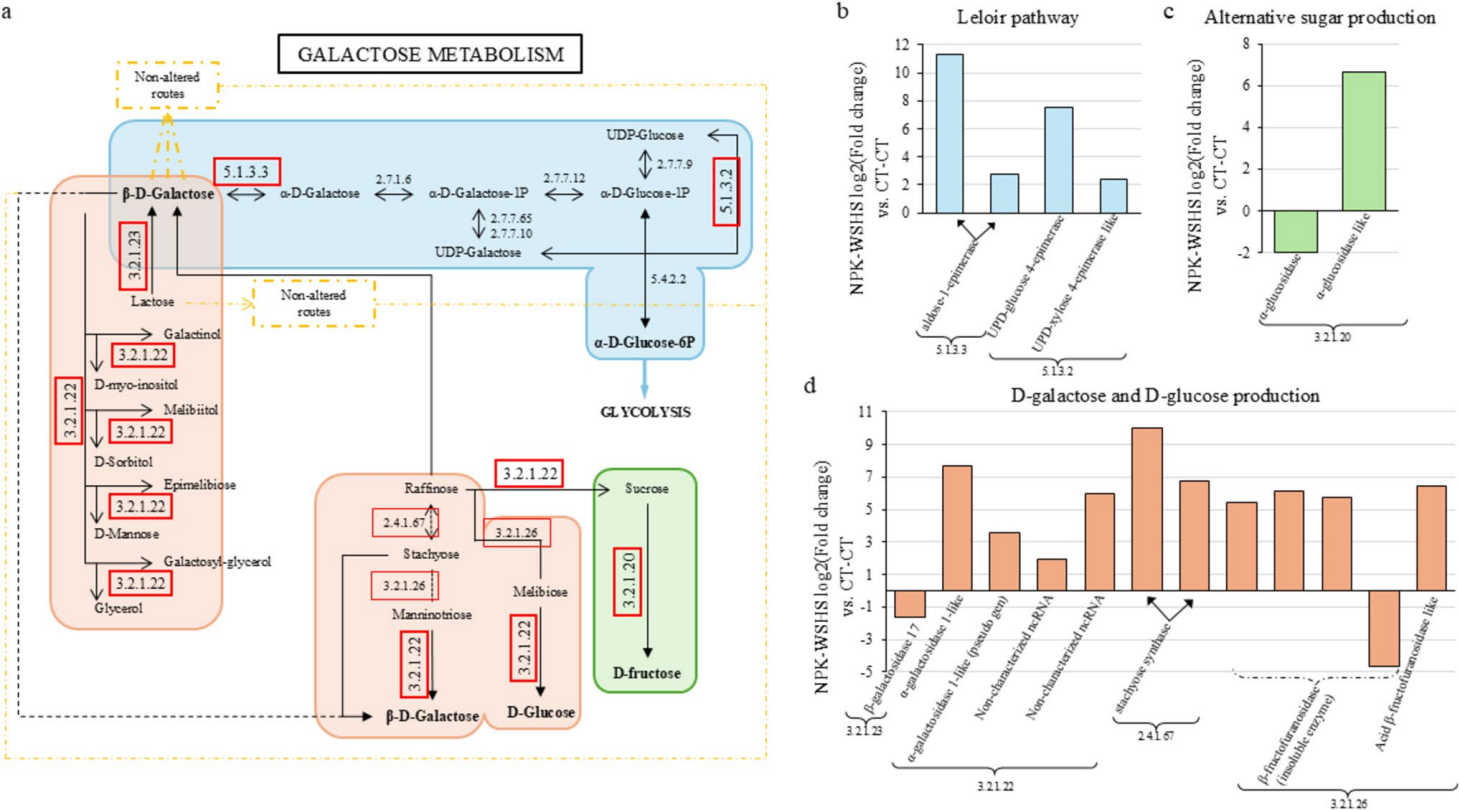
Genic expression modification of galactose metabolism pathway in plants subjected to five stresses compared to control plants. **a** Galactose metabolism pathway diagram. Numbers reference the genes involved in each conversion based on KEGG diagrams. Genes with differential expression in comparison to the control are marked with a red box, marking with a thicker line principal metabolites production. **b** Fold change values of genes involved in the Leloir pathway of plants exposed to five stresses compared to the control. **c** Fold change values of genes involved in alternative sugar production of plants exposed to five stresses compared to the control. **d** Fold change values of genes involved in D-galactose and D-glucose production of plants exposed to five stresses compared to the control

Interestingly, only two pathways exhibited significantly upregulated gene expression in stressed plants: galactose metabolism (28 genes) and taurine and hypotaurine metabolism (7 genes), involving a total of 35 genes with significant changes (adjusted *P* value of 7.28·10^–5^ and 3.00·10^–4^, respectively). Related to the galactose metabolism, the differential expression was observed in three focused routes of the whole pathway (Fig. 8a), with several genes involved in the Leloir pathway showing changes in their expression ranging from 2.41- to 11.30-fold change compared to the control (Fig. 8b). A similar pattern was observed in the expression of genes related to the production of D-galactose or alternative sugars such as fructose or glucose, with even a 7.65-fold change in their expression in comparison with the control (Fig. 8c-d).

On the other hand, downregulation was observed in a broader set of pathways, with at least 295 genes significantly repressed across 11 pathways in the most stressed plants, compared to control. The most downregulated pathways were: (1) photosynthetic activity and photosynthesis-antenna proteins (51 genes, adjusted *P* value = 1.99·10^–8^); (2) steroid biosynthesis (15 genes, adjusted *P* value = 4.1·10^–3^); and (3) ribosomal proteins (75 genes, adjusted *P* value = 6.8·10^–3^). In addition, 157 genes were differentially repressed in comparison to control plants across seven different pathways, including carbon fixation in photosynthetic organisms, porphyrin metabolism, glycosaminoglycan degradation, flavonoid biosynthesis, plant hormone signal transduction, tyrosine metabolism and ubiquinone and other terpenoid–quinone biosynthesis (Fig. 7c).

## Discussion

In this work we have demonstrated that MFSC exerts a cumulative and detrimental effect on citrus plant growth. This is clearly reflected in the citrus responses observed under combined stress conditions involving nutritional deficiencies, heat stress and water stress (Figs. 2–5; Online Resource S4). The variations in the studied parameters confirm that the combination of stresses negatively alters citrus physiological status in complex and unpredictable ways, particularly when four or more stress factors are combined, even when each individual stress alone does not impair plant performance.

The presented results show the impact of the increasing number of stresses on citrus performance, reflected first on phenotypic parameters. In the presence of three or more combined stressors, plants not only exhibit greater leaf injury, but also show reduced aerial biomass, indicating a more compromised physiological status compared to conditions with fewer than three stressors. While previous studies in herbaceous model species (Zandalinas et al. 2021; Pascual et al. 2023) have shown significant physiological alterations under MFSC, little attention has been paid to the effects of such complex stress scenarios in woody perennial crops like citrus. In particular, the combination of nutritional deficiencies with other abiotic stresses (such as water and heat stress) remains largely unexplored, despite its relevance under real agricultural conditions. This study therefore contributes valuable insights into how multiple stress interactions affect citrus physiology and growth.

This trend is repeated in ФPSII values, which are significantly reduced when three or more stresses are applied. Photosystem II, the first component of the electron transfer chain, plays a crucial role in photosynthesis, as it is the primary site for light energy capture and the initiation of electron flow (Müh and Zouni 2020). For this reason, ФPSII is used as a marker of vigour, growth, energetic status, and overall physiological activity. A decrease in ФPSII is therefore associated with reduced photosynthetic efficiency and growth (Romanowska-Duda et al. 2019).

E and gs are closely related parameters, with E controlled by changes in stomatal aperture and the differential water vapor pressure between the leaf and the atmosphere. E typically increases when stomata open, which commonly happens when plants are exposed to heat stress in order to dissipate the internal temperature (Balfagón et al. 2019a). In contrast, under drought conditions plants close their stomata to prevent water loss and transpiration is reduced (Sinha et al. 2022). When both water and heat stress are combined, the drought response predominates in citrus, leading to sustained stomatal closure. In this situation, water stress intensity plays an important role, exhibiting citrus a more drastic response with the increased intensity of the drought (Zandalinas et al. 2018a).

In addition, nitrogen and phosphorus deficiencies can reduce root hydraulic conductivity, which in turn reduces stomatal conductance and transpiration (Bouranis et al. 2012). In contrast, potassium deficiency has been demonstrated to increase stomatal conductance in different species, resulting in higher transpiration rates (Benlloch-González et al. 2008). Consequently, both E and gs are highly dependent on the specific nature of the stress conditions applied. Due to the contrasting physiological responses induced by different abiotic stresses, it was challenging to detect a consistent linear decline trend in E and gs values when citrus were subjected to one to three combined stress conditions (Fig. 3b–c). However, when four or five stresses were applied simultaneously, E and gs were significantly reduced. These data, together with the observed decline in ФPSII, reflect a more pronounced impairment of the photosynthetic machinery under the co-occurrence of a high number of stresses, even when the individual stresses alone elicit contrasting physiological responses.

The observed increase in carotenoid content under accumulating stress conditions likely reflects an adaptive response aimed at enhancing photoprotection and oxidative stress mitigation. In photosynthetic organisms, carotenoids play essential roles beyond light harvesting, including structural stabilization of pigment-protein complexes, quenching of excess energy, and detoxification of reactive oxygen species. Moreover, they serve as precursors for stress-related phytohormones involved in stress response such as ABA which modulates plant responses to environmental constraints (Uarrota et al. 2018). The accumulation of carotenoids under stress is consistent with previous findings in both microalgae and higher plants, where nutrient deficiencies and heat stress have been associated with increased carotenoid biosynthesis (Juneja et al. 2013; Zhang et al. 2017).

In contrast, chlorophyll content showed an opposite trend, decreasing with the application of multiple stressors. The decline in chlorophyll levels can be attributed to the disruption of nitrogen metabolism, given the central role of nitrogen in chlorophyll biosynthesis which has been determined by Pearson’s correlation coefficient in previous works. Indeed, a strong correlation between foliar nitrogen content and chlorophyll concentration has long been established (Bojović and Marković 2009). This antagonistic response suggests that while the plant downregulates chlorophyll production under nutritional depletion, water and heat stress, it simultaneously enhances carotenoid accumulation to safeguard the remaining photosynthetic apparatus and maintain cellular homeostasis. Chlorophyll content decrease with the increased number of stresses was not intense. As it has been previously mentioned, nitrogen is necessary for this pigment biosynthesis, decreasing its concentration under nitrogen deficiency (Bojović and Marković 2009). However, this chlorophyll content decrease was not observed with the same intensity under phosphorus or potassium deficiency, either under water or heat stress. Among the six experimental groups, different stress combinations which could or could not include nitrogen deficiency were included (Fig. 1), leading to a situation of a linear and slight chlorophyll content decrease with the stress accumulation.

The significant rise in MDA levels under the accumulation of five stress factors points to intensified lipid peroxidation and ROS production, highlighting a threshold beyond which oxidative damage becomes more evident (Pascual et al. 2023). This increase coincides with the enhancement of the antioxidant capacity, suggesting that the plant activates its defence systems in response to higher oxidative damage. This behaviour is consistent with the known physiological traits of the Carrizo citrange genotype, which has demonstrated a rapid and efficient antioxidant response under stress. Interestingly, despite the application of five simultaneous stressors, the extent of MDA accumulation was relatively moderate. This could be attributed to the robust antioxidant machinery of Carrizo, capable of buffering oxidative damage more effectively than other citrus genotypes (Vives-Peris et al. 2017). These results emphasize the role of genetic background in shaping the oxidative stress response and suggest that Carrizo may possess a degree of resilience that helps mitigate the cumulative impact of multifactorial stress.

Phytohormones are crucial molecules of plant responses to individual and combined stress conditions, acting through complex signaling networks that modulate physiological and molecular acclimation processes (Jing et al. 2024). Among them, ABA is one of the most important hormones in the abiotic stress response (Gómez-Cadenas et al. 2015), playing a central role in both rapid physiological acclimation, such as stomatal closure, and long-term transcriptional and metabolic reprogramming under stress combinations (Zandalinas et al. 2016a). Under nutritional deficiencies, plants tend to accumulate ABA especially in roots, probably to promote root development and water/nutrient foraging (Schraut et al. 2005; Vysotskaya et al. 2008). Its relevance in stress resilience is further supported by evidence showing that ABA-deficient *Arabidopsis thaliana* mutants exhibit higher susceptibility and mortality under heat stress compared to wild-type plants, largely due to impaired stomatal regulation via ABA-mediated kinase signaling cascades (Suzuki et al. 2016; Zandalinas et al. 2016a). Consistent with these findings, ABA levels in our study increased progressively with the number of applied stress factors, becoming significantly elevated in plants subjected to the most severe combinations. This pattern supports the central role of ABA in orchestrating plant responses under multifactorial stress conditions. Notably, the accumulation of PA, a primary catabolite of ABA, followed a similar trend, reinforcing the importance of an enhanced ABA turnover in increasingly stressed plants (Balfagón et al. 2019b, 2020).

JA is often involved in interactions with other plant hormones like ABA, SA or IAA (Wang et al. 2020). Rather than acting independently, JA operates within a broader signaling network, whose output varies according to the nature and intensity of the stress. For example, JA and ABA often act synergistically under drought stress, but this relationship may shift under different stress contexts (Wang et al. 2020). In plants exposed to heat stress applied individually, JA content has been observed not to change in a significant way (Balfagón et al. 2019a; Pascual et al. 2023). Under nutrient deficiency, JA biosynthesis genes are upregulated in rice, yet total JA levels often remain stable, with only certain derivatives showing an increase (Kamali et al. 2025). In our study, JA concentration progressively increased when plants were subjected to combined stress conditions, exhibiting also an exceptional increase under four stresses. This specific multifactorial stress scenario included a stress combination based on the application of phosphorus and potassium deficiency in combination with water and heat stress (PK-WSHS) in the four stresses experimental group. This specific stress combination induced a high increase of JA concentration not induced by the rest of the stress combinations included in this group, leading to an increase of JA concentration in this group that was not significant compared to the control situation. This sheds light on the unpredictability of the plant response to multifactorial stress: the plant response depends on each specific stress combination, although this response presents an accumulative pattern with the accumulated stresses, confirmed by the progressive increase observed under five stresses.

SA has been increasingly recognized for its role in plant tolerance to abiotic stresses as salinity, drought, heat, and heavy metals (Emamverdian et al. 2020; Rai et al. 2020). SA contributes to stress tolerance through the activation of antioxidant defenses, accumulation of osmolytes, and interaction with other hormonal pathways (Song et al. 2023; Yang et al. 2023). Moreover, exogenous application of SA in controlled doses has been shown to improve stress resilience across various plant species and stress combinations (Galani et al. 2016; Zhang et al. 2020; Sampedro-Guerrero et al. 2024). In our study, SA levels increased moderately in plants subjected to up to four stressors but did not exhibit a pronounced rise. This subdued response may be linked to the well-documented antagonism between JA and SA pathways, where increased JA accumulation can suppress SA signaling (Wang et al. 2020), potentially limiting SA biosynthesis or stability under certain stress combinations.

IAA concentration exhibited a high and punctual increase under one stress condition. However, with the stress accumulation, plants did not show a significant increase of IAA content compared to the control situation. Antagonistic relationships with other phytohormones, such as ABA, JA or SA, which presented a more marked increase with the accumulated stresses, could explain this situation. In contrast, plants could be prioritizing other phytohormones’ accumulation in front of IAA under complex stress scenarios because of their protective efficiency or importance in stress response.

The transcriptomic data suggests that galactose metabolism plays a relevant role in the response of citrus plants to multifactorial stress. Specifically, the upregulation of genes associated with this pathway in the most affected plants may indicate a metabolic adjustment aimed at coping with energy demands and cellular damage. Galactose metabolism is known to contribute to energy generation, cell wall biosynthesis (Gross and Pharr 1982) and stress responses (Sengupta et al. 2015; Shahbazy et al. 2020) aligning with the observed transcriptomic trends. In citrus, the accumulation of soluble sugars such as fructose or glucose, has been previously linked to the mitigation of oxidative damage and leaf injury under stress conditions (Balfagón et al. 2022a; Terán et al. 2025), suggesting that the transcriptional activation of genes in this pathway could be part of a broader protective response.

Interestingly, among the various pathways involved in galactose metabolism, the upregulation was focused on the genes involved in: (1) Leloir pathway; (2) its precursor production (D-galactose); (3) D-glucose production; and (4) D-fructose production (Fig. 8). The Leloir pathway is the primary metabolic route in galactose metabolism. It is responsible for the conversion of β-D-galactose to α-D-glucose-6P, a metabolite that feeds directly into glycolysis and central energy metabolism and can be easily produced from glucose (Sellick et al. 2008). The enhanced expression of genes driving this conversion may reflect a strategic metabolic rerouting to ensure energy availability under stress. Additionally, the observed activation of glucose and fructose biosynthesis is consistent with their roles not only as mobile sugars supporting sink tissues (Geiger 2020), but also as osmoprotectants and signaling molecules involved in stress perception and response (Terán et al. 2025). In particular, glucose is the main metabolic energy source in plants, being also the principal compound of cellulose and a signaling molecule (Geiger 2020).

Conversely, the transcriptomic profile also reflects a suppression of various energy-demanding pathways, a common response to prolonged or intense stress exposure. The downregulation of photosynthesis-related genes suggests a decline in photosynthetic activity, likely as a consequence of both stomatal closure and photodamage. This interpretation is reinforced by the parallel decreases observed in physiological parameters such as ФPSII, E, and gs (Fig. 3a–c), pointing to functional impairment of the photosynthetic apparatus.

Furthermore, the repression of genes involved in the steroid biosynthesis pathway may indicate a shift in secondary metabolism under stress. Steroids are secondary metabolites which can be classified into seven major groups, such as phytosterols, brassinosteroids or steroidal alkaloids (Gunaherath and Gunatilaka 2014). Among all the genes involved in this pathway, those downregulated were mainly involved in phytosterol synthesis which are compounds of the plant cell membrane lipid bilayer and control the membrane fluidity and permeability (Gunaherath and Gunatilaka 2014). This finding aligns with the increased MDA levels and antioxidant capacity exhibited by these plants (Fig. 4), which reflect a higher lipid peroxidation suffered by membranes.

Finally, the strong repression of ribosomal protein gene expression suggests a deliberate downscaling of protein synthesis machinery, likely as a means of conserving energy and limiting non-essential metabolic activity (Lafontaine and Tollervey 2001). This form of translational control is a known response under resource-limiting conditions and supports the notion of a coordinated stress response prioritizing survival over growth.

Taken together, the current study based on Carrizo citrange responses to MFSC highlights the fact that the accumulation of abiotic stresses, particularly nutritional deficiencies, water and heat stress, has non-predictable and detrimental effects on citrus growth, especially when three or more stresses are simultaneously applied. Stressed plants exhibited altered physiological and metabolic parameters, including decreased photosynthetic activity, increased oxidative damage, and significant changes in phytohormone profiles. These physiological disruptions were accompanied by transcriptomic shifts that revealed the downregulation of growth- and energy-intensive pathways, while selectively enhancing metabolic routes related to energy production, particularly through the Leloir pathway and soluble sugar accumulation. Notably, the impact of stress was found to intensify non-linearly beyond a certain threshold, with a marked aggravation from three simultaneous stress factors onward. Moreover, plants can present specific responses under specific stress conditions, as it was observed related to parameters such as leaf damage, E, gs, particular phytohormone profiles, or unique genic expression regulation. Despite the fact that plants tend to present an accumulative stress response with the increased number of stresses, it is important to consider that specific responses can be induced by determined stress combinations, elucidating the importance of studying the MFSC and the plants’ response to each stress scenario.

These findings, obtained under novel and agronomically meaningful stress combinations, offer new perspectives on how citrus plants cope with multiple concurrent constraints, revealing responses that cannot be inferred from studies in herbaceous species or from isolated stress factors alone. As such, our findings help to understand and manage citrus resilience in the face of increasingly complex environmental challenges.

## Supplementary Information

Below is the link to the electronic supplementary material.

Supplementary file1 (PDF 400 KB)

## Data Availability

Data will be made available on reasonable request.

## Abbreviations

E: Transpiration
gs: Stomatal conductance
JA: Jasmonic acid
MDA: Malondialdehyde
MFSC: Multifactorial stress combination
ФPSII: Photosystem II quantum yield
SA: Salicylic acid
