# Supplementary material for: Integrated analysis of multifactorial stress combination impact on citrus plants
Source: Planta. 2025 Nov 5;262(6):145. doi: 10.1007/s00425-025-04866-z (PMC12589267; doi:10.1007/s00425-025-04866-z)
Supplement: Supplementary file 1 — Supplementary file1 (PDF 400 KB) [file 425_2025_4866_MOESM1_ESM.pdf]

Integrated analysis of Multifactorial Stress Combination impact on citrus plants

Lledó Rodríguez-Azorín[1], Aurelio Gómez-Cadenas[1], María F. López-Climent[1*], Vicente Vives-Peris[1*].

[1]*Department of Biology, Biochemistry and Natural Sciences, Universitat Jaume I, Avda. Sos Baynat s/n, 12071, Valencian Community, Castelló de la Plana, Castelló, Spain*

*Corresponding authors: Vicente Vives-Peris and María Fernanda López-Climent; E-mail addresses: vvives@uji.es and mcliment@uji.es. Phone numbers: +34 964728101 and +34 964729414

Journal: Planta

Online resource S1. Irrigation solutions composition at 10X concentration. CT = control condition; N = nitrogen deficiency condition; P = phosphorus deficiency condition; K = potassium deficiency condition; combined letters = combination of different nutritional deficiencies.

Irrigation solution 10X		
**Condition**	**Reagents**	**(g/L)**
**CT**	KNO_3	40
	$MgSO_4 \cdot 7\ H_2O$	40
	H_3PO_4 72%	9.3
	Nutrishell	4
	$Ca(NO_3)_2$	82
**N**	K_2SO_4	34.48
	$MgSO_4 \cdot 7$ H2O	40
	H_3PO_4 72%	9.3
	Nutrishell	4
	$CaCl_2$	55.4
**P**	KNO_3	40
	$MgSO_4 \cdot 7\ H_2O$	40
	Nutrishell	4
	$Ca(NO_3)_2$	82
**K**	$(NH_4)_2SO_4$	26.2
	$MgSO_4 \cdot 7\ H_2O$	40
	H3PO4 72%	9,3
	Nutrishell	4
	$Ca(NO_3)_2$	82
**NK**	$MgSO_4 \cdot 7\ H_2O$	40
	H_3PO_4 72%	9,3
	Nutrishell	4
	$CaCl_2$	55.4
**NP**	K_2SO_4	34.48
	$MgSO_4 \cdot 7\ H_2O$	40
	Nutrishell	4
	$CaCl_2$	55.4
**PK**	K_2SO_4	34.48
	$MgSO_4 \cdot 7\ H_2O$	40
	Nutrishell	4
	$Ca(NO_3)_2$	82
**NPK**	$MgSO_4 \cdot 7\ H_2O$	40
	Nutrishell	4
	$CaCl_2$	55.4

Online resource S2. The thirty-two combinations based on the applied abiotic stress combination. CT = control condition; N = nitrogen deficiency condition; P = phosphorus deficiency condition; K = potassium deficiency condition; WS = water stress condition; HS = heat stress condition; combined letters = combined stresses.

**Combinations 1 – 4**		**Combinations 5 – 8**		**Combinations 9 – 12**		**Combinations 13 – 16**	
1st phase stress	2nd phase stress	1st phase stress	2nd phase stress	1st phase stress	2nd phase stress	1st phase stress	2nd phase stress
**CT**	CT	**N**	CT	**P**	CT	**K**	CT
	WS		WS		WS		WS
	HS		HS		HS		HS
	WSHS		WSHS		WSHS		WSHS
**Combinations 17 – 20**		**Combinations 21 – 24**		**Combinations 25 – 28**		**Combinations 29 – 32**	
1st phase stress	2nd phase stress	1st phase stress	2nd phase stress	1st phase stress	2nd phase stress	1st phase stress	2nd phase stress
**NP**	CT	**NK**	CT	**PK**	CT	**NPK**	CT
	WS		WS		WS		WS
	HS		HS		HS		HS
	WSHS		WSHS		WSHS		WSHS

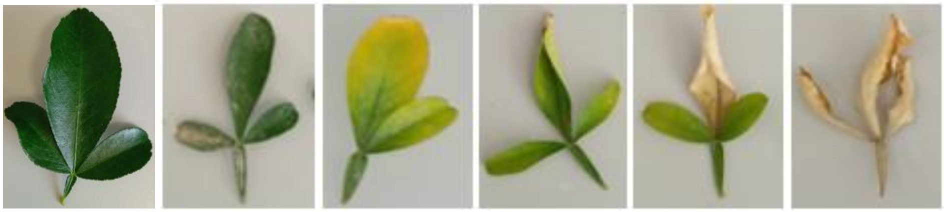

0 1 2 3 4 5

Online Resource S3. Standard on which leaf damage level was based.

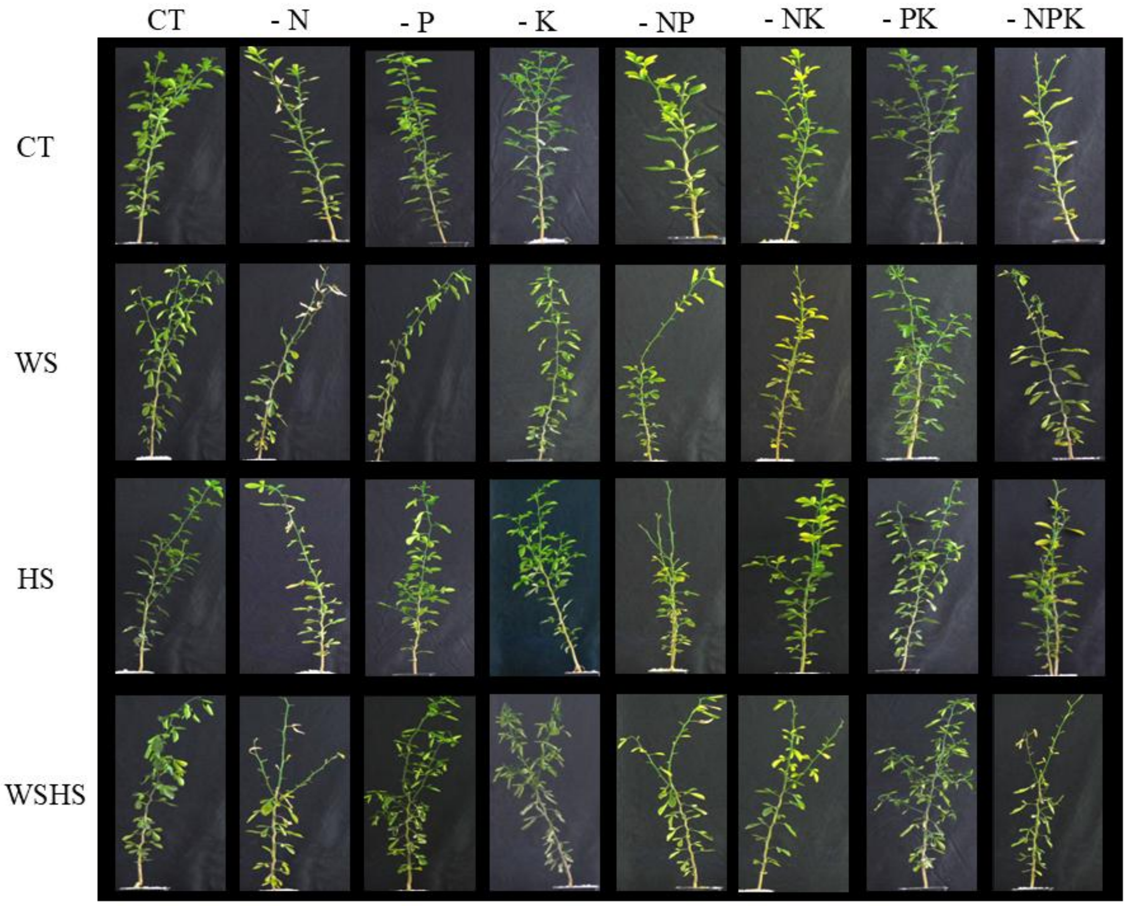

Online Resource S4. Picture of the most representative citrus plant exposed to a stress combination on the sampling day. In the horizontal axis, abbreviations of the first phase groups: CT = control; N = nitrogen deficiency; P = phosphorous deficiency; K = potassium deficiency; combine letters = combined deficiencies. In the vertical axis, abbreviations of the second phase groups: CT = control group; WS =water stress group; HS = heat stress group; combined letters = combined stresses.

Online resource S5. Chlorophyll A (ChlA) and chlorophyll B (ChlB) content depending on the number of stresses in plants subjected to Multifactorial Stress Combination. Data refer to mean values ± SE. Statistical analysis was performed by ANOVA test using *P*-value = 0.05 comparing all the groups simultaneously. Different letters denote significative difference between groups in "ANOVA" column. An additional statistical analysis was performed by Student's t-test using *P*-value = 0.05. Ref. = reference.

	**Chl a content**				**Chl b content**			
**Stresses number**	**Mean (µg/g FW)**	**SE**	**ANOVA**	**T-student**	**Mean (µg/g FW)**	**SE**	**ANOVA**	**T-student**
**0**	1803.50	40.96	a	Ref.	575.35	14.78	a	Ref.
**1**	1765.97	92.96	a	0.8627	615.46	33.36	a	0.6079
**2**	1733.40	96.56	a	0.8225	628.44	38.24	a	0.6683
**3**	1619.45	114.84	a	0.6210	607.89	52.82	a	0.8490
**4**	1626.49	148.56	a	0.6105	583.44	59.43	a	0.9534
**5**	1496.38	76.11	a	0.0253	530.50	330.98	a	0.2614

Online Resource S6. Phytohormone content values of citrus exposed to Multifactorial Stress Combination depending on the number of applied stresses. ABA = abscisic acid; PA = phaseic acid; SA= salicylic acid; IAA = indole acetic acid; JA = jasmonic acid; EE = standard deviation. Data refer to mean values ± SE. Statistical analysis was performed by ANOVA test using *P*-value = 0.05 comparing the six groups simultaneously for each hormone. Different letters denote significative difference between groups in "ANOVA" column. An additional statistical analysis was performed by Student's t-test using *P*-value = 0.05. Ref. = reference.

**Hormone**	**Stresses number**	**Mean (ng/g FW)**	**SE**	**ANOVA**	**T-student**
**ABA**	0	19.66	1.74	a	Ref.
	1	159.01	43.47	a	0.181
	2	287.05	56.72	a	0.146
	3	296.58	53.62	a	0.112
	4	252.47	58.05	a	0.099
	5	221.70	11.72	a	0.000
**PA**	0	92.38	6.29	a	Ref.
	1	217.59	37.18	a	0.161
	2	294.07	46.46	a	0.179
	3	323.06	48.65	a	0.150
	4	279.77	62.47	a	0.209
	5	366.69	48.49	a	0.005
**SA**	0	67.30	7.85	a	Ref.
	1	53.10	5.12	a	0.235
	2	58.06	3.62	a	0.411
	3	67.96	5.38	a	0.968
	4	90.31	5.43	a	0.077
	5	119.97	12.19	a	0.022
**IAA**	0	2.46	1.48	a	Ref.
	1	8.14	1.09	a	0.037
	2	10.98	1.81	a	0.135
	3	11.55	1.77	a	0.104
	4	13.84	2.55	a	0.071
	5	7.56	3.20	a	0.222
**JA**	0	31.02	1.74	a	Ref.
	1	43.82	12.19	a	0.643
	2	145.21	54.55	a	0.505
	3	241.47	88.17	a	0.455
	4	576.72	297.71	a	0.439
	5	246.26	23.78	a	0.001

Online Resource S7. Principal Component Report obtained in PCA performed to analyse the differences on leaf damage, total carotenoids and total chlorophylls content, shoot fresh weight, ΦPSII, gs, E, MDA content, antioxidant capacity, ABA content, JA content, SA content, PA content and IAA content of citrus plants exposed to Multifactorial Stress Combination depending on the number of stresses (**Fig. 6**).

	**PC1**	**PC2**
**ΦPSII**	-0.195	0.185
**E**	0.251	0.409
**gs**	0.248	0.410
**Leaf damage**	0.0615	-0.480
**Shoot fresh weight**	-0.0231	0.376
**Total carotenoids**	0.439	-0.0371
**Total chl**	0.357	0.0605
**ABA**	0.382	-0.101
**JA**	0.383	-0.0216
**SA**	0.113	0.212
**IAA**	0.184	-0.350
**PA**	0.0525	-0.246
**MDA**	0.156	0.0513
**Antioxidant capacity**	0.383	-0.111
